# Migraine misdiagnosis as a sinusitis, a delay that can last for many years

**DOI:** 10.1186/1129-2377-14-97

**Published:** 2013-12-12

**Authors:** Jasem Y Al-Hashel, Samar Farouk Ahmed, Raed Alroughani, Peter J Goadsby

**Affiliations:** 1 Department of Neurology, Ibn Sina Hospital, P.O. Box 25427, Safat 13115 Kuwait City, Kuwait; 2 Department of Medicine, Faculty of Medicine, Health Sciences Centre, Kuwait University, Kuwait City, Kuwait; 3 Department of Neurology and Psychiatry, Al-Minia University, Minia, Egypt; 4 Division of Neurology, Amiri Hospital, P.O. Box 1661, Qurtoba 73767 Kuwait City, Kuwait; 5 Division of Neurology, Dasman Diabetes Institute, P.O. Box 1180, Dasman 15462 Kuwait City, Kuwait; 6 Wellcome Trust Clinical Research Facility, Kings College Hospital, London, UK

**Keywords:** Migraine misdiagnosis, Sinus headache

## Abstract

**Background:**

Sinusitis is the most frequent misdiagnosis given to patients with migraine.

Therefore we decided to estimate the frequency of misdiagnosis of sinusitis among migraine patients.

**Methods:**

The study included migraine patients with a past history of sinusitis. All included cases fulfilled the International Classification of Headache Disorders, 3^rd^ edition (ICHD-III- beta) criteria. We excluded patients with evidence of sinusitis within the past 6 months of evaluation. Demographic data, headache history, medical consultation, and medication intake for headache and effectiveness of therapy before and after diagnosis were collected.

**Results:**

A total of 130 migraine patients were recruited. Of these patients 106 (81.5%) were misdiagnosed as sinusitis. The mean time delay of migraine diagnosis was (7.75 ± 6.29, range 1 to 38 years). Chronic migraine was significantly higher (*p* < 0.02) in misdiagnosed patients than in patients with proper diagnosis. Medication overuse headache (MOH) was reported only in patients misdiagnosed as sinusitis. The misdiagnosed patients were treated either medically 87.7%, or surgically12.3% without relieve of their symptoms in 84.9% and 76.9% respectively. However, migraine headache improved in 68.9% after proper diagnosis and treatment.

**Conclusions:**

Many migraine patients were misdiagnosed as sinusitis. Strict adherence to the diagnostic criteria will prevent the delay in migraine diagnosis and help to prevent chronification of the headache and possible MOH.

## Background

Migraine continues being an underdiagnosed condition [1] because it can be accompanied by symptoms commonly associated with other causes of facial pain [2,3]. Many patients visit their general practitioner because of their headache and, in many cases, a proper diagnosis and treatment may take years [4,5].

“Sinusitis” may constitute one of the most commonly confusing clinical presentation of migraine [6], probably because cranial autonomic symptoms are common in migraine [7] based on activation of the trigeminal-autonomic reflex [8]. Headaches located in the frontal, supraorbital, or infraorbital region are sinus headaches [9]. These headaches are usually recurrent, non-seasonal, and unassociated with fever, localized tenderness, or erythema [10].

Migraine-associated alterations in trigeminal and/or autonomic activity may explain nasal and ocular symptoms in migraine. For example, “sinus” symptoms in migraine have been hypothesized to arise from activation of the trigeminal-autonomic reflex, which is mediated by a circuit of trigeminal afferents and parasympathetic efferent that innervate the lacrimal glands and the nasal mucosa [11].

This study aimed to estimate the frequency of misdiagnosis of sinusitis among patients with migraine headache who fulfilled the diagnostic criteria according to the International Classification of Headache Disorders, 3^rd^ edition (ICHD-III- beta) criteria [12].

## Methods

This retrospective study included 130 male and female migraine patients aged above 12 years with history of sinusitis. Every headache was assigned a diagnosis based ICHD-III-beta [12].

Exclusion criteria included radiographic evidence of sinus infection, the occurrence of fever, or purulent nasal discharge associated with their headaches within the past six months of evaluation. Patients who were unable to give reliable information about their medical history and headache characteristics or have incomplete medical files were excluded from the study (n = 17).

### Patient identification

The data were collected from a hospital-based cohort of headache patients referred to both Mubarak and Ibn Sina Hospitals, Kuwait. We examined the medical files of all patients diagnosed with headache who were registered between 2010 and 2012. The records were examined and standardized data collection forms were completed retrospectively by the study group.

### Clinical data

The diagnosis of migraine was made during face to face interview with headache specialist. Demographic characteristics, headache frequency, duration, and associated headache symptoms were recorded for each patient. Patients were asked about the onset of their headache, how long it took them to receive a correct diagnosis (latency of diagnosis) and what different physicians they had consulted prior to the current consultation. Results of previous diagnostic investigation including brain and sinus imaging were retrieved also.

Patients were asked about their use of medical and surgical treatments for headache before migraine diagnosis and to rate the effectiveness of each treatment before and after diagnosis on a 4-point scale: [13]

1. Very effective – complete and long-lasting relief

2. Effective –partial and/or short-lasting relief

3. Ineffective

4. Headache worsened.

### Data analysis

All analyses were performed using SPSS 19 for Windows. Simple descriptive statistical tests (Mean and Standard deviation) were used to describe the numerical values of the sample. Frequency and percentage were used to describe the non-numerical values of the sample. The significance of the differences between the patients with proper and misdiagnosis was determined using a chi-squared test for non- numerical variables. *P* < 0.05 was defined as statistically significant.

The study received the approval of the local ethic committee, and all the patients signed the appropriate informed consents.

## Results

Table 1 describes the demographic and characteristic data of 130 migraine patients.

**Table 1:** **Demographic data and characteristic of all migraine patients (*n*** **= 130) according to ICHD-III-beta**

**Variable**	**Mean ± SD/no. (%)**
**Age**	35.88 ± 9.87
**Duration of headache**	10.22 ± 7.60
**Sex**	
Male	30 (23.1%)
Female	100 (76.9%)
**Symptoms referred to sinus area**	
Sinus pain	99 (76.2%)
Sinus congestion	78 (60%)
Nasal congestion	72 (55.4%)
**Investigations looking at the sinuses**	
Sinus x ray	58 (44.6%)
Sinus CT	21 (16.2%)
Endoscopy	3 (2.3%)
**Neuroimaging**	
MRI brain	44 (33.8%)
CT brain	6 (4.6%)
MRI cervical spine	2 (1.5%)

### Symptoms

The symptoms referred to the sinus areas were: sinus pain (76.2%), sinus pressure (60%) and nasal congestion (55.4%). Most of our patients had at least one investigation looking at the sinuses. Thirteen patients (10%) showed thickened sinus mucosa in CT sinuses. Many patients had at least one performed neuroimaging test and all of them were normal (Table 1).

### Headache diagnosis

We found that 106 (81.5%) of our patients had been misdiagnosed as sinusitis. Chronic migraine was significantly higher (*p* < 0.0001) in misdiagnosed patients and medication over use headache (MOH) reported only in patients misdiagnosed as sinusitis (Table 2).

**Table 2:** Comparison of headache profiles according to ICHD-III between migraine patients with proper diagnosis and those with misdiagnosis

**Type of headache**	**Migraine patients with proper diagnosis (n = 24)**	**Migraine patients misdiagnosed as sinusitis (n = 106)**	**P value**
Migraine without aura [1.1]	13 (54.2%)	43 (40.6%)	0.2
Migraine with aura [1.2]	8 (33.3%)	22 (20.8%)	0.1
Chronic migraine [1.3]	3 (12.5%)	30 (28.3%)	0.02*
Medication over use headache [8.2]	0	11 (11.4%)	

*Significant.

### Misdiagnosis results

The mean duration of headache in misdiagnosed patients was 11.15 ± 7.85 (range 2 to 40 years) and the mean time between the first attack of headache and the diagnosis of migraine was 7.75 ± 6. 29 (range 1 to 38 years). 59/106 (56.6%) of them had consulted a primary care physician, and 47/106 patients (43.4%) assessed by otorhinolaryngology specialist before the diagnosis of migraine was made.

Thirteen patients (12.3%) of them had prior “sinus surgery” based on suspected lesions on CT sinuses and 93 (87.7%) received medical treatment for sinusitis. Both surgical and medical treatment were ineffective in most of the patients. Of those who received ineffective medical or surgical treatment, 73 (69%) were more satisfied in 2–4 months after initiating anti-migraine treatment (topiramate, propranolol, amitriptyline or sodium valproate) after their proper diagnosis (Table 3).

**Table 3:** Effective of management prior and after migraine diagnosis

**Variable**	**No. (%)**
**Effective of management prior migraine diagnosis**	
Surgical (no = 13)	
Very effective – complete and long-lasting relief	0/13
Effective –partial and/or short-lasting relief	3/13 (22.1%)
Ineffective	9/13 (69.2%)
Headache worsened	1/13 (7.7%)
Medical (no = 93)	
Very effective – complete and long-lasting relief	0/93
Effective –partial and/or short-lasting relief	14/93 (15.1%)
Ineffective	65/93 (69.8%)
Headache worsened	14/93 (15.1%)
**Effective of treatment after migraine diagnosis (no = 106)**	
Very effective – complete and long-lasting relief	73/106 (68.9%)
Effective –partial and/or short-lasting relief	22/106 (20.8%)
Ineffective	8/106 (7.5%)
Headache worsened	3/106 (2.8%)

## Discussion

Our study included 130 patients with migraine-type headache according to 3^rd^ edition (ICHD-III-beta). We found that 81.5% of them were misdiagnosed and managed as sinusitis. The similarity of sinusitis symptoms and migraine complicates the diagnostic evaluation process. Although both historical and new data show that nasal symptoms frequently accompany a migraine, these symptoms are not required by the ICHD-III-beta diagnostic criteria for a migraine.

Our data are in agreement with the study of Schreiber and colleagues [6] which included approximately 3000 patients with a history of self-described or physician-diagnosed “sinus” headache and they determined that 80% of patients met ICHD criteria for migraine. Our data are also in agreement with the other previous studies [3,9,14,15] which reported that “sinus headache” is one of the most commonly reported terms used in combination with a migraine diagnosis and most patients presenting with a “sinus headache” may not actually have a rhino sinusitis associated headache.

Migraine can be mistaken for rhinosinusitis because of similarity in location of the headache and the commonly accompanying nasal autonomic symptoms. The presence or absence of purulent nasal discharge and/or other features diagnostic of acute rhinosinusitis help to differentiate these conditions [12]. In order to properly establish a diagnosis of migraine, it is essential to know the ICHD criteria and apply these criteria in clinical practice.

We demonstrated that chronic migraine was significantly higher in patients misdiagnosed with sinusitis. MOH was reported only in those patients. A delay in the diagnosis of migraine led to chronification of the headache and transformation, in some cases, into MOH.

We found that the diagnosis of migraine was delayed in more than 80% of our cohort up to 38 years. Eross and colleagues [15] similarly found that their patients waited 25.3 years (longest of 62 years) prior to the correct diagnosis. Previous studies showed this as well [4,5]. The diagnostic delay in our cohort could be explained by the presence of sinus pain, sinus congestion and nasal discharge during headache attacks. These symptoms have been reported with previous studies which concluded that presence of autonomic symptoms during migraine attacks often leads to confusion and incorrect diagnosis of sinusitis [16,17]. The ICHD criteria do not highlight the presence of cranial autonomic symptoms in the disorder, or perhaps more usefully comment upon them. This may help general practitioner and otorhinolaryngology specialist to be aware of the phenotyping overlap.

The majority of our patients had at least one investigation looking at the sinuses which were all normal. This result is similar to previous results which demonstrated that patients with “sinus headache” did not have findings suggestive of sinusitis on endoscopy or CT scan [14] and over 50% of them were diagnosed with migraine later [18]. These unnecessary investigations increase time delay to obtain the correct diagnosis and management [19].

We demonstrated that 56% of the misdiagnosed patients had consulted a primary care physician and 44% of them an otorhinolaryngology specialist before the diagnosis of migraine was made. We are in agreement with Foroughipour and colleagues [17] who studied 58 patients with the diagnosis of sinusitis made by a primary care physician. After comprehensive otorhinolaryngologic and neurologic evaluation, the final diagnoses was migraine in 68% of the patients. Furthermore, our study demonstrated that the misdiagnosed patient received either medical in 87.7%, or surgical treatment in 12.3% of them without relieve of their symptoms in 84.9% and 76.9% respectively. However, migraine headache improved in 68.9% after proper diagnosis and treatment. These results are similar to that of Foroughipour and colleagues [17] who reported that recurrent antibiotic therapy was received by 66% patients and therapeutic nasal septoplasty was performed in 16% of the patients with a final diagnosis of migraine.

An appropriate recognition of migraine in patients who complain about sinus headaches may help to minimize the suffering and unnecessary interventions, start migraine directed therapy [20] and improve quality of life [9].

## Conclusion

In conclusion, symptoms suggestive of sinusitis are frequently seen in migraine patients and may lead to delayed diagnosis and treatment of migraine. General practitioner and otorhinolaryngology specialist should be aware of the diagnostic criteria for migraine and consider it in their differential diagnosis of patients suffering from “sinusitis”. Going forward it is important to consider how best to draw attention to cranial autonomic symptoms in migraine and their place in diagnostic criteria.

## Competing interest

The authors declare that there are no conflicts of interest.

## Authors’ contributions

JAH designed the study, collected the data and revised the manuscript, SFA collected, and analyzed the data, and wrote the manuscript, RA and PJG revised the manuscript. All authors read and approved the final manuscript.

## References

[B1] Stewart WF Simon D Shechter A Lipton RB Population variation in migraine prevalence: a meta-analysis J Clin Epidemiol 1995 14 269 80 10.1016/0895-4356(94)00128-D 7869073

[B2] Cady RK Schreiber CP Sinus headache or migraine? considerations in making a differential diagnosis Neurology 2002 14 suppl 6 S10 S14 12011268 10.1212/wnl.58.9_suppl_6.s10

[B3] Diamond ML The role of concomitant headache types and non-headache comorbidities in the underdiagnosis of migraine Neurology 2002 14 suppl 6 S3 S9 12011267 10.1212/wnl.58.9_suppl_6.s3

[B4] De Diego EV Lanteri-Minet M Recognition and management of migraine in primary care: influence of functional impact measured by the headache impact test (HIT) Cephalalgia 2005 14 3 184 90 10.1111/j.1468-2982.2004.00820.x 15689193

[B5] Ryan RE Jr Pearlman SH Common headache misdiagnoses Prim Care Clin Office Pract 2004 14 395 405 10.1016/j.pop.2004.02.010 15172514

[B6] Schreiber CP Hutchinson S Webster CJ Ames M Richardson MS Pharm D BCPS; Powers C Prevalence of migraine in patients with a history of self-reported or physician-diagnosed “Sinus” headache Arch Intern Med 2004 14 1769 1772 10.1001/archinte.164.16.1769 15364670

[B7] Peter JG Lacrimation, conjunctival injection, nasal symptoms…cluster headache, migraine and cranial autonomic symptoms in primary headache disorders- what's new? J Neurol Neursurg Psychiatry 2009 14 1057 1058 10.1136/jnnp.2008.162867 19762895

[B8] May A Goadsby PJ The trigeminovascular system in humans: pathophysiological implications for primary headache syndromes of the neural influences on the cerebral circulation J Cereb Blood Flow Metab 1999 14 115 127 10027765 10.1097/00004647-199902000-00001

[B9] Dadgarnia MH Atighechi S Baradaranfar MH The response to sodium valproate of patients with sinus headaches with normal endoscopic and CT Findings Eur Arch Otorhinolaryngol 2010 14 375 379 10.1007/s00405-009-1090-9 19756679

[B10] Levine HL Setzen M Cady RK An otolaryngology, neurology, allergy and primary care consensus on diagnosis and treatment of sinus headache. A literature review Otolaryngol Head Neck Surg 2006 14 516 523 10.1016/j.otohns.2005.11.024 16500456

[B11] Akerman S Holland P Goadsby PJ Diencephalic and brainstem mechanisms in migraine Nat Rev Neurosci 2011 14 570 584 10.1038/nrn3057 21931334

[B12] Headache Classification Committee of the International Headache Society The International Classification of Headache Disorders, 3rd edition (beta version) Cephalalgia 2013 14 629 808 23771276 10.1177/0333102413485658

[B13] Rossi P Faroni J Tassorelli C Nappi G Diagnostic delay and suboptimal management in a referral population with hemicrania continua Headache 2009 14 227 234 10.1111/j.1526-4610.2008.01260.x 19222596

[B14] Mehle ME Kremer PS Sinus CT scan findings in “Sinus Headache” migraineurs Headache 2008 14 67 71 18184288 10.1111/j.1526-4610.2007.00811.x

[B15] Eross E Dodick D Eross M The Sinus, Allergy and Migraine Study (SAMS) Headache 2007 14 213 224 10.1111/j.1526-4610.2006.00688.x 17300361

[B16] Barbanti P Fabbrini G Pesare M Unilateral cranial autonomic symptoms in migraine Cephalalgia 2002 14 256 259 10.1046/j.1468-2982.2002.00358.x 12100086

[B17] Foroughipour M Sharifian SM Shoeibi A Ebdali Barabad N Bakhshaee M Causes of headache in patients with a primary diagnosis of sinus headache Eur Arch Otorhinolaryngol 2011 14 11 1593 6 10.1007/s00405-011-1643-6 21626445

[B18] Perry BF Login IS Kountakis SE Nonrhinologic headache in atertiary rhinology practice Otolaryngol Head Neck Surg 2004 14 449 452 10.1016/j.otohns.2004.01.005 15100642

[B19] Viticchi G Silvestrini M Falsetti L Lanciotti C Cerqua R Luzzi S Provinciali L Bartolini M The role of instrumental examinations in delayed migraine diagnosis Neurol Sci 2011 14 Suppl 1 S143 4 21533731 10.1007/s10072-011-0520-9

[B20] Patel ZM Kennedy DW Setzen M Poetker DM Delgaudio JM “Sinus headache”: rhinogenic headache or migraine? An evidence-based guide to diagnosis and treatment Int Forum Allergy Rhinol 2013 14 221 230 10.1002/alr.21095 23129234

